# Mitochondrial autophagy in the sleeping brain

**DOI:** 10.3389/fcell.2022.956394

**Published:** 2022-08-24

**Authors:** Sofia Mauri, Mariavittoria Favaro, Greta Bernardo, Gabriella M. Mazzotta, Elena Ziviani

**Affiliations:** Department of Biology, Centro Studi per la Neurodegenerazione (CESNE), University of Padova, Padova, Italy

**Keywords:** mitophagy, proteostasis, Parkinson’s disease, circadian rhythms, animal models

## Abstract

A significant percentage of the mitochondrial mass is replaced on a daily basis via mechanisms of mitochondrial quality control. Through mitophagy (a selective type of autophagy that promotes mitochondrial proteostasis) cells keep a healthy pool of mitochondria, and prevent oxidative stress and inflammation. Furthermore, mitophagy helps adapting to the metabolic demand of the cells, which changes on a daily basis.

Core components of the mitophagy process are PINK1 and Parkin, which mutations are linked to Parkinson’s Disease. The crucial role of PINK1/Parkin pathway during stress-induced mitophagy has been extensively studied *in vitro* in different cell types. However, recent advances in the field allowed discovering that mitophagy seems to be only slightly affected in PINK1 KO mice and flies, putting into question the physiological relevance of this pathway *in vivo* in the whole organism. Indeed, several cell-specific PINK1/Parkin-independent mitophagy pathways have been recently discovered, which appear to be activated under physiological conditions such as those that promote mitochondrial proteome remodeling during differentiation or in response to specific physiological stimuli.

In this Mini Review we want to summarize the recent advances in the field, and add another level of complexity by focusing attention on a potentially important aspect of mitophagy regulation: the implication of the circadian clock. Recent works showed that the circadian clock controls many aspects of mitochondrial physiology, including mitochondrial morphology and dynamic, respiratory activity, and ATP synthesis. Furthermore, one of the essential functions of sleep, which is controlled by the clock, is the clearance of toxic metabolic compounds from the brain, including ROS, via mechanisms of proteostasis. Very little is known about a potential role of the clock in the quality control mechanisms that maintain the mitochondrial repertoire healthy during sleep/wake cycles. More importantly, it remains completely unexplored whether (dys)function of mitochondrial proteostasis feedbacks to the circadian clockwork.

## Introduction

The circadian clock is a conserved internal timing system that allows inhabitants of planet earth, from plants and bacteria to insects and mammals, to adapt and anticipate periodic changes of the external environment. Circadian rhythms are generated by an endogenous biological system, the circadian clock, which influences most biological processes, such as rest/activity behaviour, sleep/wake cycles, feeding, body temperature fluctuations, hormonal secretion, and many others (Panda et al., 2002). They are physiological and behavioural cycles that exhibit a periodicity of about 24 h, in synchrony with the solar time, and are generated by three key components: a pacemaker that operate with an approximate 24-h period even under constant environmental conditions; input pathways (i.e. light) that synchronize the pacemaker to the environment; and output pathways that convert the molecular oscillation of clock components in overt rhythms reviewed in Patke et al. (Patke et al., 2020). In mammals, the central pacemaker is located in the suprachiasmatic nucleus (SCN), a bilateral structure in the anterior hypothalamus comprising about 20.000 clock neurons (Patke et al., 2020). Light information (“input”) is conveyed to the SCN *via* the retinohypothalamic tract, resulting in an intracellular signaling cascade, which converges on cAMP-response elements in the promoters of several key clock genes (“output”). In addition to the central pacemaker, circadian clocks are also present in many other tissues: these peripheral or “slave” oscillators are coordinated by the SCN through neurotransmitters and neuromodulators, but can also entrain to signals other than light (Dibner et al., 2010).

**FIGURE 1:**
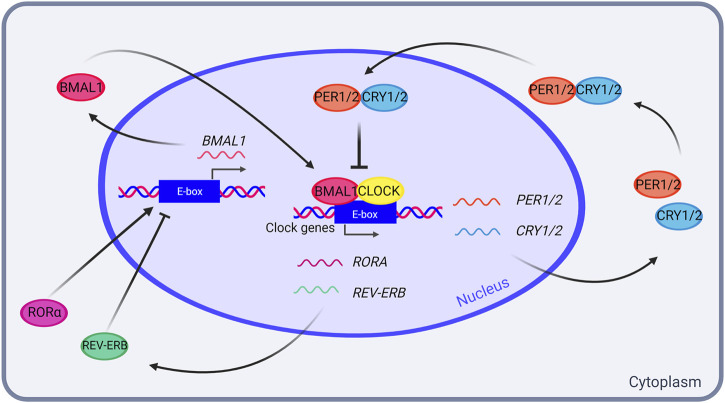
The mammalian transcriptional-translational feedback loop (TTFL). In the mammalian TTFL, transcription activators CLOCK and BMAL1 bind to the E-boxes in *PER1/2* and *CRY1/2* promoters, resulting in their transcription. PER1/2 and CRY1/2 accumulate in the cytoplasm, and form a heterodimer that enters the nucleus and inhibits the CLOCK-BMAL1 complex, thus inhibiting their own transcription in a feed-forward negative loop. In addition, CLOCK and BMAL1 also promote *RORA* and *REV-ERB* transcription, which in turn activate and inhibit *BMAL1* transcription, respectively. Created with BioRender.com.

At the molecular level, endogenous circadian oscillations are generated by a self-sustained and cell-autonomous evolutionary conserved transcriptional-translational feedback loop (TTFL), in which positive elements promote the rhythmic transcription of the negative elements that, in turn, inhibit the activity of the positive elements (Dunlap, 1999). The mammalian primary TTFL involves the interaction between the transcriptional activators CLOCK and BMAL1, and the negative elements formed by PERIOD (PER1, PER2 and PER3) and CRYPTOCHROME (CRY1 and CRY2) reviewed in Cox and Takahashi (Cox and Takahashi, 2019). The CLOCK/BMAL1 heterodimer binds to E-box elements present in the promoter of Per1/2 and Cry1/2 genes, triggering their expression. PER/CRY complexes translocate into the nucleus where they inhibit their own transcription by directly associate to CLOCK/BMAL1. CLOCK/BMAL1 also induce the expression of the nuclear receptors REV-ERBα and REV-ERBβ (REV-ERBα/β), which act in negatively regulating Bmal1 transcription (reviewed in Cox and Takahashi (Cox and Takahashi, 2019) (Figure 1).

Circadian rhythmicity has a very relevant role in everyday life, it is therefore not surprising that circadian disruptions are closely related to the development of diseases like cancer, cardiovascular disease, obesity, diabetes and sleep disorder reviewed in Xie et al. (Xie et al., 2019). Of note, the activity of the circadian system changes significantly across the lifespan, and rhythmic outputs, in particular sleep/wake cycle, are strongly impaired in aged individuals (Hood and Amir, 2017). Many of the age-related disturbances in the circadian functions resemble the circadian disturbances observed in neurodegenerative disorders such as Alzheimer’s Disease (AD), Parkinson’s Disease (PD) and Huntington’s Disease (HD), although the severity and timing of their onset in patients are different compared to their occurrence during healthy aging (Hood and Amir, 2017). In PD in particular, 60–90% of patients present sleep disturbances that appear at early stage of the disease, and profoundly impact the quality of life (Barone et al., 2009; Raggi et al., 2013; Raggi and Leonardi, 2013). Moreover, diurnal fluctuation in symptoms associated with PD, such as motor symptoms, visual performance and responsiveness to dopaminergic treatments, have been described (Struck et al., 1990), leading to the hypothesis of a circadian control on the expression of clinical features of PD.

In the investigation of the molecular mechanism linking circadian rhythms to neurodegeneration in PD, mitochondria appear as an interesting suspect. These double membrane organelles are essential for cell life, representing the major source of cellular ATP and performing many functions, not least overseeing the metabolic demand of the cell. Mitochondria are highly dynamic, and can adapt their activity to meet the different bioenergetic demands of the cell through cycles of mitochondrial fission and fusion. Nearly every aspect of mitochondrial function depends on mitochondrial dynamics, including mechanisms of mitochondrial quality control that have evolved to maintain the mitochondrial repertoire healthy, and prevent oxidative stress and inflammation. Two proteins, PINK1 and Parkin, which mutations are linked to juvenile forms of PD, belong to the core machinery that surveys mitochondrial proteostasis, by orchestrating the selective removal of damaged mitochondria via autophagy. In the process of mitochondrial autophagy (hereafter called mitophagy), the mitochondrial network undergoes important morphological changes that depend on the coordinated activity of fission and fusion events. Importantly, while many studies support the hypothesis of a clock-dependent regulation of mitochondrial dynamic, very little attention has been paid to explore whether mitochondrial proteostasis, mitophagy in particular, undergoes circadian oscillation, how this is generated, and whether this is a cell-autonomous or non-autonomous mechanism. Hints towards the hypothesis of a clock-dependent regulation of general proteostasis come from the observation that inessential byproducts and proteotoxic compounds, such as β-amyloids, accumulate during the active phase, and are degraded by the proteasome while sleeping (Kang et al., 2009; Varshavsky, 2012; Xie et al., 2013; Shokri-Kojori et al., 2018; Varshavsky, 2019). Moreover, an extensive metabolic rewiring occurs during sleep in order to adjust to the different requirement and availability of energy supply. In this context, a critical role is played by autophagy, which contributes to nutrients and cellular homeostasis. While the rhythmicity of the autophagic process has indeed been demonstrated by several studies (Ma et al., 2011; Ryzhikov et al., 2019), the connection between the circadian clock and mitochondrial autophagy in particular has not been fully explored, but a similar daily-based proteostatic mechanism for mitochondrial homeostasis can be predicted.

In the following chapters we will summarize recent advances in the field of mitophagy, and focus our attention on a potentially important aspect of mitochondrial proteostasis: the implication of the circadian clock and sleep homeostasis.

### Molecular mechanisms of mitochondrial autophagy

Quality control mechanisms have evolved to maintain healthy cells, and they are crucial for cellular function. They mainly relay on the proteolitic activity of the ubiquitin proteasome and lysosome-autophagy system, two fundamental degradative systems that cooperate to oversee protein and organelle quality control. Among these mechanisms of quality control, of particular relevance are those that regulate the quality of the mitochondrial repertoire. Mitochondria represent the major site of cellular ATP biosynthesis, and perform essential metabolic functions. These double membrane organelles can however become professional killers, and major sources of oxidative stress. Thus, impaired mechanisms of mitochondrial quality control can pose a threat for cell function and survival, especially when these organelles are not functioning properly.

**FIGURE 2:**
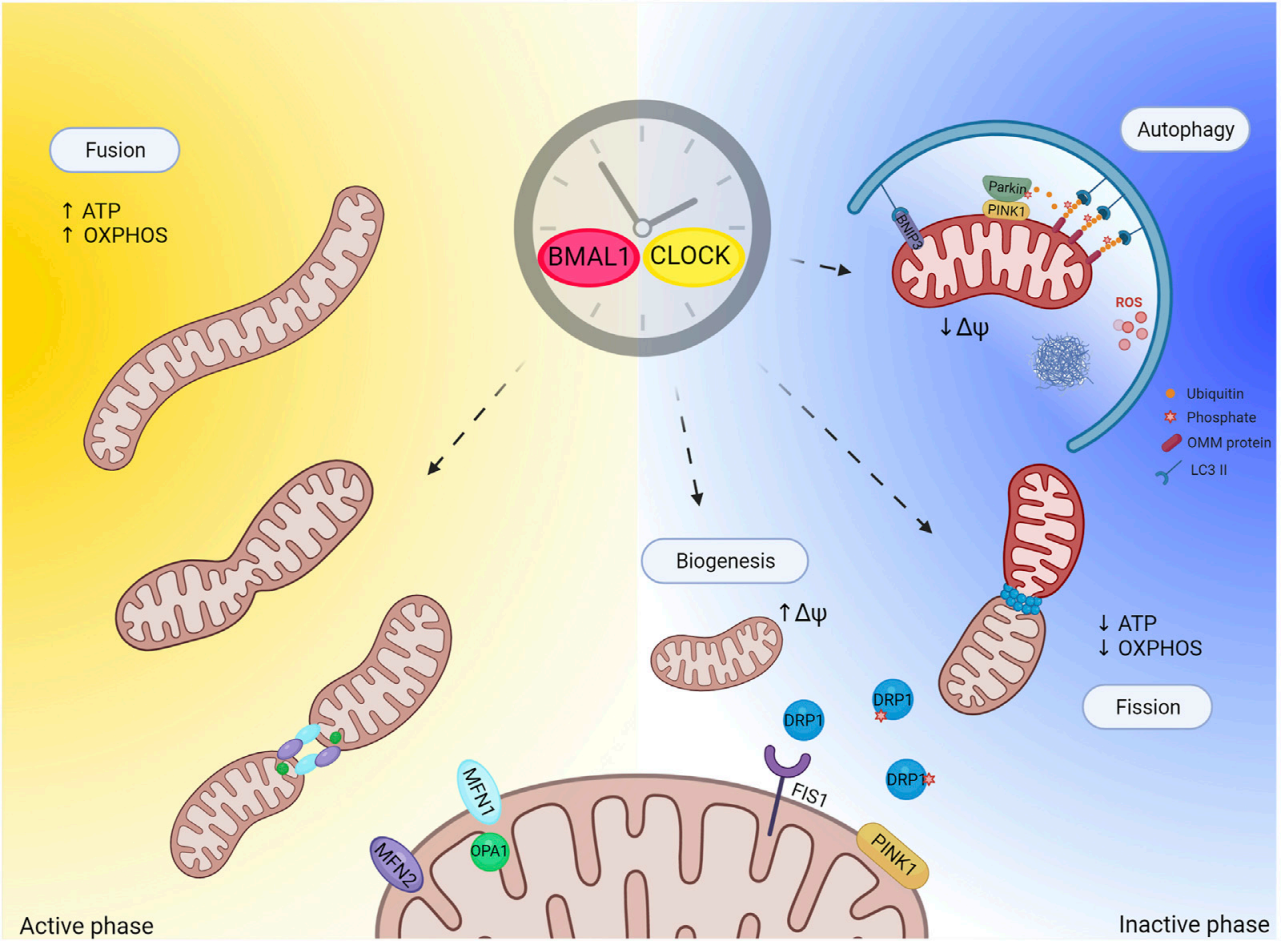
Circadian clock and regulation of mitochondrial quality control. Figure depicts hypothetical regulation of mitochondrial quality control by the clock. Mitochondria quality control (mitophagy and biogenesis in particular) strongly relays on mitochondrial dynamic (fusion and fission events). Mitochondrial fission generates uneven daughter units, one unit exhibiting increased mitochondrial membrane potential and high probability to fuse, while the other has decreased mitochondrial membrane potential and is degraded by autophagy. The circadian clock regulates mitochondrial fission to allow mitochondrial adaptation to daily metabolic changes. This regulation might also extend to mitochondria quality control. Importantly, one proposed function of sleep (inactive phase) is prevention of oxidative stress build up and degradation of toxic compounds. In light of this, one potential physiological role of sleep during the inactive phase could be to promote mitochondrial quality control as antioxidant response. Created with BioRender.com.

Protein kinase PINK1 and E3 ubiquitin ligase Parkin contribute to mitochondrial quality control by regulating the selective removal of damaged mitochondria via mitophagy. Upon mitochondrial insults that induce depolarization of mitochondrial membrane potential (MMP), protein kinase PINK1 is stabilized on the surface of a subset of mitochondria (i.e. those that are damaged, and loose their membrane potential) (Narendra et al., 2008; Narendra et al., 2010), it phosphorylates Ubiquitin (Kane et al., 2014; Koyano et al., 2014), the E3 Ubiquitin-ligase Parkin (Kim et al., 2008; Shiba-Fukushima et al., 2014; McWilliams et al., 2018a), and Parkin substrates on the outer mitochondrial membrane (OMM) (Chen and Dorn, 2013), and promotes activation and mitochondrial recruitment of Parkin (Trempe et al., 2013; Wauer and Komander, 2013; Kane et al., 2014; Kazlauskaite et al., 2014; Ordureau et al., 2014; Kumar et al., 2015; Seirafi et al., 2015; Wauer et al., 2015). Activated Parkin ubiquitinates a large subset of OMM-resident proteins (Sarraf et al., 2013; Bingol et al., 2014), leading to the recruitment of Ub-binding receptors, such as OPTN (Heo et al., 2015) and NDP52 (Lazarou et al., 2015), and promoting the formation of the autophagosome, a double-membrane phagophore that wraps around the selected organelle, and delivers its cargo to the lysosome for degradation (Figure 2). These evidences indicate that PINK1 and Parkin interact functionally in a linear pathway that promotes mitophagy, with PINK1 operating upstream of Parkin, and elegant *in vivo* studies in flies fully support this hypothesis (Clark et al., 2006; Park et al., 2006; Yang et al., 2006). There are however important variations on the theme. For example, in cardiac myocytes, Parkin is recruited to depolarized mitochondria and activates mitophagy in the absence of PINK1 (Kubli et al., 2015), indicating that alternative mechanisms of Parkin activation and mitophagy operate in the absence of PINK1. Moreover, PINK1/Parkin-dependent mitophagy has been described in the absence of MMP depolarization, for example following proteotoxic stress (Jin and Youle, 2013; Burman et al., 2017), and more recently, upon specific induction of mitochondrial Ca^2+^ oscillation (Yu et al., 2021). These studies suggest that the PINK1/Parkin pathway might not be that linear, and more importantly, that it can be activated upon physiological stimuli controlled by Ca^2+^ homeostasis, which do not necessarily culminate in mitochondrial damage or mitochondrial ROS production.

Intuitively, the mitochondrial network needs to fragment to allow efficient phagophore formation around the cargo. Indeed, expression of dominant-negative Drp1 to inhibit mitochondrial fission (Twig et al., 2008), or stabilization of pro-fusion proteins Mfn1 & Mfn2 to exacerbate mitochondrial fusion, prevents mitophagy (Tanaka et al., 2010). Elongated and fused mitochondria are protected from mitophagy, likely because their size does not allow proper engulfment (Gomes et al., 2011; Rambold et al., 2011), while promotion of mitochondrial fission facilitates mitophagy, although with some important exceptions (Song et al., 2015; Yamashita et al., 2016). Beside the size issue, fission events allow segregating dysfunctional mitochondria from the mitochondrial network, and sorting out oxidized mitochondrial proteins and mutant mtDNA copies to concentrate the damage into one daughter mitochondrion that is subsequently eliminated via autophagy (Twig et al., 2008; Twig and Shirihai, 2011). In parallel, mitochondrial biogenesis compensates for mitochondrial loss, and maintains the mitochondrial repertoire stable. Importantly, the mitochondrial network is not made *de novo* but rather recruits and imports new proteins to the organelle with subsequent division by fission to generate uneven daughter units. In this scenario, one unit exhibits increased MMP and high probability to fuse, while the other has decreased MMP, loses its pro-fusion capacity, and is eliminated by autophagy (Twig et al., 2008). Thus, mitochondrial biogenesis and mitophagy are intimately correlated, and mitochondrial fission appears to be important for both.

PINK1 directly contributes to mitochondrial fission in several ways: by disrupting the AKAP1-PKA complex (Pryde et al., 2016), which is known to phospho inhibit pro-fission protein Drp1 at Ser 637, and by directly phosphorylating Drp1 on Ser 616 to boost Drp1 mitochondrial recruitment and fission activity (Han et al., 2020). Drp1 is selectively recruited to mitochondria in the proximity of PINK1/Parkin, suggesting that mitochondrial division occurs at sites where the PINK1/Parkin-dependent mitochondrial clearance program is initiated (Buhlman et al., 2014) (Figure 2). Once again, elegant studies performed in flies support the hypothesis of a genetic interaction between PINK1/Parkin and mitochondrial fission in that promotion of mitochondrial fission rescues the aberrant mitochondrial and locomotor phenotype associated to PINK1 KO and Parkin KO flies, while promotion of mitochondrial fusion exacerbates the pathological phenotype (Poole et al., 2008). These evidences indicate that promotion of mitochondrial fission in these flies prevents the aberrant phenotype associated to PINK1 and Parkin loss, presumably by facilitating elimination of dysfunctional mitochondria.

Despite intense investigation and compelling evidences highlighting the relevance of the PINK1/Parkin pathway for mitophagy under physiological conditions, such as those controlling mitochondrial partitioning upon cell division (Mishra and Chan, 2014), stem cell renewal (Ito et al., 2016), and synapses function (Palikaras and Tavernarakis, 2020), the importance of this pathway *in vivo* is still debated. A proteomic-based analysis of radiolabeled mitochondria proved that the mitochondrial turnover is affected by the loss of Parkin and PINK1 *in vivo* in flies (Vincow et al., 2013). However, basal mitophagy measured with fluorescent-based mitophagy reporters mito-QC and mt-Keima, seems to be only slightly affected in PINK1 KO flies (also supported by studies in mice (McWilliams et al., 2018b)), even if aberrant mitochondria are known to accumulate in the fly tissues, particularly in the muscle of the thorax, which incidentally is among those tissues in which mitolysosomes are not detected at all (Lee et al., 2018). This unexpected result put into question the physiological relevance of the PINK1/Parkin pathway *in vivo.* There are however several possible explanations for this lack of effect. It is possible that the mitophagic reporters are simply not sensitive enough to detect small physiological mitophagic events, which *in vivo* might predominately occur in a piece meal fashion (McLelland et al., 2014). Another possibility is that PINK1 KO animals need to be subjected to some sort of mitochondrial stress to develop a readable mitophagic defect. Works in flies support this hypothesis in that PINK1 and Parkin KO flies develop defective mitophagy only when they are aged (Cornelissen et al., 2018) or in response to hypoxic exposure and rotenone treatment (Kim et al., 2019). Alternatively, it is possible that PINK1/Parkin-independent mitophagic pathways are activated during development to compensate for PINK1 loss. Recent advances in the field allowed discovering several cell-specific PINK1/Parkin-independent mitophagy pathways, which are activated in response to specific physiological stimuli to promote mitochondrial proteome remodelling, for example during maturation of erythrocytes (Schweers et al., 2007; Kundu et al., 2008), neuronal differentiation (Esteban-Martinez et al., 2017; Ordureau et al., 2021), maturation of muscle cells (Sin et al., 2016), and white adipose tissue transition (Wrighton, 2016; Vamos et al., 2022). In support of the role of compensatory mechanisms are also recent publications showing that mitochondrial quality control pathways are intertwined, so that activation of one specific pathway can compensate for loss of another (Koentjoro et al., 2017; Towers et al., 2021).

Another intriguing hypothesis is that the occurrence of the PINK1/Parkin mitophagy pathway changes on a daily basis depending on the metabolic demand of the cell, which also changes during the day, and most predictably between the active and inactive phase of the organism. In this respect, if we hypothesize that a specific mitophagy pathway occurs during the inactive phase (i.e. during the night for diurnal animals like humans and flies), loss of it might undergo unnoticed if the readout in real time is performed during the active phase. This could partially explain why the fluorescent mitophagy reporters did not record a readable difference in basal mitophagy between WT and PINK1 KO flies, while the proteomic approach proved to be more effective.

### Circadian regulation of mitochondrial dynamic

The notion that mitochondrial dynamics is implicated in metabolic adaptation to changes in nutrient influx, such as those that presumably occur between day and night, is not novel and supported by many studies (Manella and Asher, 2016; Leong, 2018; Schmitt et al., 2018). Indeed, mitochondria accommodate different energy demands by complex morphological adaptations that dictate their function, and in particular tubular mitochondria perform at higher respiration capacity and ATP synthesis (Gomes et al., 2011), while fragmented mitochondria exhibit lower spare respiratory capacity and low ATP levels (Bach et al., 2003). Thus, it is perhaps not surprising that mitochondrial morphology exhibits a circadian rhythmicity to fulfil predictable changes in nutrient availability. However, elegant studies performed *in vivo* in mice liver showed that mitochondrial dynamic is controlled by circadian rhythmicity in a fashion that is independent of nutrient intake. In particular, mice with liver specific ablation of clock gene Bmal1 exhibit disrupted rhythmicity in the gene and protein level of pro-fission Fis1 and in the mitophagic genes and proteins Pink1 and Bnip3, accompanied with enlarged and swollen mitochondria, dysfunctional oxidative metabolism and ATP synthesis. Importantly, this effect of Bmal1 ablation is independent of nutrient availability. Thus, in BMAL1 deficient cells, protein levels of Fis1, PINK1 and BNIP3 fail to accumulate in anticipation of the upcoming feeding phase, leading to disrupted mitochondrial fission and quality control that are presumably required for the metabolic remodeling that anticipates the feeding phase. Notably, promotion of mitochondrial fission rescues the aberrant metabolic and circadian phenotype associated to BNIP3 KO animals, supporting the hypothesis that mitochondrial dynamic and quality control can signal back to re-establish a functional clock (Kohsaka et al., 2014; Jacobi et al., 2015). Similar results are also observable in the heart tissue (Kohsaka et al., 2014; Li et al., 2020), where BMAL1 seems to directly regulate mitochondrial fission and mitophagy through transcriptional activation of mitophagy receptor BNIP3 (Li et al., 2020).

Further studies consolidated the hypothesis of a clock-dependent regulation of mitochondrial fission in different tissues and cells, and add another layer of complexity by demonstrating that post translational modifications of pro-fission protein DRP1, phosphorylation (Schmitt et al., 2018) and ubiquitination (Yuan et al., 2021) in particular, regulate mitochondrial dynamic in anticipation of predictable metabolic clues, although mechanistically this relationship has not been fully depicted. The reciprocal correlation between the clock and DRP1-dependent fission is corroborated by numerous evidences: cells lacking DRP1 do not display circadian oscillation of oxidative phosphorylation and ATP level; in PER1/2 KO cells lacking a functional circadian clock, the mitochondrial network remains fragmented, and ATP levels and ox/phos stop oscillating; and finally, pharmacological or genetic inhibition of mitochondrial fission significantly increases period length, and abrogates circadian oscillation in Bmal1 and Per1/2 gene expression (Schmitt et al., 2018). The latter in particular has been interpreted as an important evidence of mitochondrial dynamic signalling back to modulate circadian metabolism, although the potential involvement of core components of the fusion machinery (i.e. Mfn1/2, OPA1) has not been taken into account (Schmitt et al., 2018).

Besides BMAL1, other regulators of the circadian clock have been recently implicated in mitochondrial quality control: for example, Rev-erb-α deficiency in muscles results in impaired mitochondrial biogenesis and enhanced mitophagy (Woldt et al., 2013), whereas Clock mutants show defects in mitochondrial clearance in cardiac myocytes (Rabinovich-Nikitin et al., 2021). These correlative studies further support the hypothesis of a circadian regulation of mitochondrial proteostasis, although the molecular mechanism has not been fully elucidated. Importantly, because clock genes seem to have additional widespread physiological functions beside circadian regulation, we cannot exclude the possibility that the effects on mitochondrial quality control might depend on these pleiotropic functions. Nevertheless, the fact that mitophagy gene Bnip3 is rhythmically expressed in the mouse liver (Jacobi et al., 2015) and that BMAL1 specifically drives Bnip3 expression (Li et al., 2020), points to a direct role of clock proteins in the circadian regulation of mitochondrial quality control.

### Daily basis regulation of mitochondrial proteostasis and physiological importance

It is well known that the SCN degenerates during the aging process. Healthy aging is also associated with a physiological decrease in the number of intrinsically photoreceptive ganglion cells (ipRGCs) in the retina, whose connections deliver photic information to the SCN to synchronize the clock. These evidences can partially explain why older adults develop circadian dysfunction and sleep disturbances. Nevertheless, patients with neurodegenerative diseases experience more pronounced circadian rhythm dysfunction compared to age-matched counterpart, and disturbance in the sleep-wake cycle is perhaps the most common non-motor symptom found in up to 80% PD patients. Interestingly, in the retina of PD patients the number of ipRGCs is reduced compared to age-matched counterpart (Ortuno-Lizaran et al., 2018), and the presence of an impaired pupillary light reflex demonstrates that retinal ganglion cells functionality is also affected (Joyce et al., 2018). Moreover, PD patients often exhibit visual or retinal impairments. These deficiencies support the hypothesis of a reduced light detection in PD patients, which could be at the base of the defective synchronization of the circadian clock. Another hint towards a potential defective synchronization of the circadian clock in PD patients comes from the evidence that levels of melatonin, a hormone secreted by the pineal gland implicated in the synchronization of the clock, are affected in PD patients (Nassan and Videnovic, 2022). Importantly, melatonin has been shown to be neuroprotective in PD (Singhal et al., 2012), and in mitochondria in particular, this hormone appears to offer protection against oxidative stress (Tan et al., 2016a; Tan et al., 2016b) and to affect autophagy and mitophagy, although with conflicting results (reviewed in Wu et al., 2021; Chen et al., 2021; Jiang et al., 2021; Xie et al., 2021).

Several correlative studies support the hypothesis of a causal link between neurodegeneration in PD and aberrant circadian rhythms, with both neurotoxin and genetic animal models of PD reproducing the defective circadian pattern observed in patients (Table 1). However, although sleep disorders and circadian disruption are extremely common and precede the onset of the cognitive decline and motor symptoms of PD by many years, it is still unclear whether circadian disruption can influence the pathogenesis of PD, and therefore be a risk factor for developing the disease by contributing to neurodegeneration.

**TABLE 1:** Animal models in Parkinson’s Disease.

Model	Genetic manipulation	Intervention	Circadian phenotype
Monkey (*Macaca fascicularis and Macaca mulatta*)		MPTP-injection	Abnormally elevated night-time activity; decreased daytime activity (Panda et al., 2002)
Mouse	alpha-synuclein overexpression (ASO mice)	MPTP-injection	Reduced locomotor activity (Patke et al., 2020)
			Fragmented circadian rhythmicity; reduced firing rate of SCN neurons during the day (Dibner et al., 2010)
	Spontaneous deletion in the Uch-l1 gene (gad mice)		Destabilized circadian locomotor activity rhythms (Dunlap, 1999)
	Inactivation of mitochondrial transcription factor A (Tfam) in DA Neurons (MitoPark)		
			Fragmented circadian activity rhythms (Cox and Takahashi, 2019)
Sprague-Dawley rats		6-OHDA-injection	Altered expression of clock genes in the striatum (Xie et al., 2019)
		Rotenone-injection	Decreased amplitude and increased fragmentation of locomotor activity and body temperature circadian rhythms (Hood and Amir, 2017)
*Drosophila melanogaster*	TP-αS, A53T overexpression		Altered bout number and length; circadian locomotor periodicity shift with aging in TP-αS flies (Barone et al., 2009)
	d*Mul1* loss of function		Increased total locomotor activity; increased length of the period of locomotor activity (Raggi and Leonardi, 2013)
	d*park* loss of function		Weakened circadian rhythms in locomotor activity; reduced morning and evening peaks of activity (Raggi et al., 2013)
			Absence of circadian locomotor anticipation in the morning in LD conditions (Struck et al., 1990)
			Increased total locomotor activity; increased length of the period of locomotor activity (Raggi and Leonardi, 2013)
	d*Pink1* loss of function		Arrhythmic (Raggi et al., 2013)
			Lower total locomotor activity, increased sleep during the day (Xie et al., 2013)
			Absence of circadian locomotor anticipation in the morning in LD conditions (Struck et al., 1990)

In the investigation of the potential molecular mechanism linking PD to disruption of circadian oscillation, loss of mitochondrial proteostasis is a potential suspect. The retina is a region of high-metabolic demand of the CNS, and mitochondrial dysfunction has been linked to retinal pathology (Barot et al., 2011), thus aberrant mechanism of mitochondrial quality control, mitochondrial proteostasis in particular, might be of particular relevance for the function of the retina, and therefore for the efficient synchronization of the clock. Recently, different studies in the vertebrate retina suggest that cone photoreceptors undergo daily basis mitochondrial turnover, with mitochondrial pro-fusion protein MFN2 and mitophagy protein PINK1 displaying similar daily rhythms with peaks in the dark phase, in anti-phase with that of DRP1, indicative of cycles of mitochondrial fusion, fission and quality control at different times during the day (Chang et al., 2018). Similarly, analysis of mitochondrial morphological changes in cone photoreceptors of zebrafish showed that at night mitochondrial biogenesis is active in these cells, while during the day, mitochondria are more associated with the ER and autophagosomes, supportive of ongoing mitophagy in this condition (Giarmarco et al., 2020). This effect on the mitochondrial turnover seems to be more significantly affected by light exposure rather than by the circadian oscillators (except for the oscillation of DRP1, which persists also in complete darkness), suggesting that efficient mitochondria turnover is probably the result of both cell-autonomous and non-autonomous mechanisms (Chang et al., 2018).

Mitophagy and autophagy levels have been recently measured in different cells of the mammalian eye, using the mito-QC mitophagy reporter (McWilliams et al., 2016) and a GFP-mCherry-LC3 (McWilliams et al., 2018b) mouse models respectively. This comparative analysis demonstrates that macroautophagy occurs to varying degrees in all tissues analyzed within the eye, and that mitophagy is not directly correlated with autophagy levels, but it is a process highly dependent on context *in vivo* (McWilliams and Ganley, 2019). The application of the mito-QC model in the study of mitochondrial homeostasis in relation to the circadian clock would be of great interest to evaluate how mitophagy levels change at different time points, and whether they display a circadian rhythm under constant darkness conditions or they are affected by light exposure.

It is currently unclear whether enhancement of autophagy/mitophagy may serve to protect neurons during neurological disorders or whether it can boost neuronal damage. For instance, sleep deprivation, which is used to study the homeostatic component of sleep, has been recently linked to excessive pathological enhancement of autophagy and mitophagy (Dai et al., 2021). It is possible that enhancement of mitophagy may initially serve as a protective mechanism to limit oxidative stress, which is known to build up upon sleep deprivation (Vaccaro et al., 2020), but becomes pathological if sleep deprivation persists. Thus, simple enhancement of a pleiotropic process as vital as proteostasis may bring more harm than good in neurodegenerative conditions, if this is not paralleled by compensatory biogenesis. Interestingly, sleep deprivation increases the brain expression of the clock gene *Per2* in a time-of-day dependent manner (Curie et al., 2015), and the knock-out of one or more clock genes leads to the alteration of sleep homeostatic markers (Franken, 2013), supporting the hypothesis of a crosstalk and a reciprocal regulation between circadian rhythmicity and sleep homeostasis (Borbely et al., 2016). Sleep is orchestrated by the complex interplay between sleep-wake homeostasis, which represents sleep debt, and circadian rhythms, which determines the periodicity in wake/sleep propensity (Borbely et al., 2016); both components need to be explored in more details to clearly dissect whether they might independently affect mitophagy, or if their mutual influence may ultimately impinge on the beneficial or detrimental effect of mitophagy.

## Discussion

The circadian clock controls mitochondrial dynamic to allow mitochondrial adaptation to predictable metabolic daily changes. Mechanistically, the relationship between the clock and mitochondria dynamic has not been fully depicted, but cell-autonomous oscillation of mitochondrial fission that depend on the expression of clock core components, seems to be of crucial importance (Jacobi et al., 2015; Schmitt et al., 2018; Li et al., 2020).

Post-mitotic cells like neurons are thought to be particularly sensitive to disruptions in mechanisms of mitochondrial dynamics. In these cells, mitochondrial fission exerts pro-survival effects by many means (Kageyama et al., 2012), not least by supporting uncoupled respiration to reduce oxidative stress, and by promoting mitochondrial quality control (Twig et al., 2008; Goldsmith et al., 2022). Being high metabolic demanding, long-lived cells, with a complex architecture, neurons strongly depend on mechanisms of mitochondrial quality control, which mainly relay on mitochondrial dynamic and the proteolytic activity of the lysosome-autophagy system. Indeed, promoting mitochondrial fission or autophagy rescues the pathological phenotype of animal models of neurodegeneration, presumably by enhancing the degradation of misfolded proteins and dysfunctional organelles, which are known to accumulate and induce intracellular damage. While many studies investigate the effect of potentiating protein and organelle homeostasis to scavenge intracytoplasmic neurotoxic aggregates and defective mitochondria, very little attention has yet been paid to explore the potential link between alteration in mitochondrial homeostasis and (in)stability of core components of the circadian clock in neurodegenerative conditions, PD in particular. Disturbed sleep and circadian dysfunction is an important issue in PD, and among the earliest non-motor symptoms identified in patients, even before PD is diagnosed. Thus, sleep disturbances, as well as non-motor symptoms in general, which also seem to be linked to the clock (Schuch et al., 2018; Bolsius et al., 2021), offer a pre-symptomatic window for the treatment of this disease, allowing earlier intervention. Importantly, sleep and circadian abnormalities in PD are mirrored in several animal models, and these models are therefore useful tools to dissect the potential link between PD-associated circadian defects and the clock machinery. Flies, like human, are diurnal animals, in which the molecular mechanisms orchestrating circadian rhythm and sleep homeostasis have been extensively characterized. Thus, flies are predicted to become a key model to investigate this topic in the near future.

It is currently unclear whether sleep and circadian dysfunction are causes or consequences of neurodegenerating DA neurons, and how mitochondrial physiology fits into this picture. Nevertheless, elucidating the mechanism underlying the putative relationship between mitochondrial homeostasis, the circadian clock, and neurodegeneration will help establishing whether a cause-effect relationship exists between the circadian and mitochondrial defective phenotypes observed in patients with neurodegenerative diseases, and if treating circadian disturbance in patients with neurodegenerative diseases may have therapeutic implication.
